# Rubella IgM epidemiology in the pre-rubella vaccination era in Uganda

**DOI:** 10.1186/s12879-020-4928-9

**Published:** 2020-03-12

**Authors:** Fred Bagenda, Edgar Mugema Mulogo, Richard Onyuthi Apecu, Anette Kisakye, Benard Toliva Opar

**Affiliations:** 1 Department of Community Health, Mbarara University of Science and Technology, P O Box, 1410 Mbarara, Uganda; 2 Department of Medical Laboratory Science, Mbarara University of Science and Technology, P O Box, 1410 Mbarara, Uganda; 3 World Health Organization, Mbarara, Uganda; 4 Uganda National Expanded Programme of Immunization, Mbarara, Uganda

**Keywords:** Rubella, IgM, Suspected measles cases, Associated factors, Uganda

## Abstract

**Background:**

Control of Rubella and Congenital Rubella Syndrome using vaccination has shown great success in the America’s. Uganda is due to introduce the Rubella vaccine however the magnitude of transmission is not well documented. Therefore this study was done to determine IgM sero-prevalance for Rubella in order to help monitor vaccine effectiveness post introduction of the vaccine in routine vaccination programme.

**Methods:**

A retrospective review of suspected measles cases data for the reporting period January 2007 to December 2016 in Uganda was Don*e. rubella* IgM testing was done on 15,296 of the cases and the data was analyzed using STATA version 13.

**Results:**

In total 15,296 cases were tested and 4255 (27.8%) tested positive and among females aged 15-49 years 88 out of 322 (27%) tested positive. The age distribution range was 0–80 years, rubella IgM positivity was reported in all the 15 regions of Uganda and throughout the ten year period in every month. Age group 5–15 years had OR 2.5 *p*-value < 0.001 of being rubella IgM positive compared to age < 5 years and testing measles IgM negative OR 6.3 p-value < 0.001.

**Conclusion:**

Rubella is endemic in Uganda and although rubella IgM positivity is highest in the age 5-15 years even the younger, older and women of reprodutive age are affected. This means the risk of Congenital Rubella Syndrome is high hence the need to introduce the rubella vaccine for infants and pregnant mothers and continued surveillance to enhance its control.

## Background

Rubella and measles infections are similar in presentation with fever and rash as the main Symptom*s. rubella* is caused by an RNA rubella virus and is usually a mild condition with measles causing a more severe clinical manifestation and common among children. If vertical transmission occurs during the first trimester of pregnancy the virus causes miscarriage, still birth or Congenital Rubella Syndrome (CRS) which has teratogenic effects that include deafness, blindness, congenital heart disease and mental retardation [1, 2].

Although the exact prevalence of CRS is not know it is estimated that more than 100,000 cases were born especially in the developing countries in 2015 [3, 4]. The prevalence of rubella among all people in Africa is estimated at 52.9 to 97.9% and that of pregnant women in Africa has been reported to range from 54.1 to 95.2% a public health importance despite the reported high immunity in the younger children [4–8]. The general rubella-specific IgG sero-prevalance in pre-vaccination era in Africa is comparable with other regions in Southern America, India and Europe before vaccination. However, the level of natural immunity in these studies is lower than the immunity currently reported in Europe; this might be due to on-going vaccination programmes in developed countries [6, 9].

Rubella is a vaccine preventable disease and the rubella vaccine contains a live attenuated virus that is effective in combination as trivalent with measles and mumps or bivalent with measles. The WHO global measles rubella initiative and strategic plan targets to eliminate measles and rubella by 2020 however the WHO African region has not yet set a target. In the WHO American region due to intensified vaccination against measles and rubella there has been no indigenous measles case since 2002 and no rubella case since 2009. WHO declared the American region to have eliminated rubella and CRS in 2009 and 2015 respectively. In the United States of America evidence from data collected and analyzed on the epidemiology and geno types of rubella and CRS showed that rubella had been eliminated by 2004 due to an intensive rubella vaccination programmes which had been started in 1969 [3, 10–12]. Rubella vaccine is widely available and is part of the routine vaccination programs in the developed countries but this is not the case in many African counties including Uganda. In Uganda there is limited or no pro-active process of routinely screening pregnant mothers for rubella and their possible vaccination.

Although the Uganda National Expanded Programme of Immunization (UNEPI) intends to introduce the rubella containing vaccine by 2019 there is no systematic documentation for epidemiological information up to 2012 pre vaccine era apart from information on the genotypes [13, 14]. In Uganda, the Uganda Virus Research Institute (UVRI) EPI laboratory is part of the WHO network of laboratories that has its own database while the EPI program also has a more detailed measles case based surveillance epidemiological database within the routine Integrated Disease Surveillance and Response (IDSR). At the UVRI, Expanded Programme of Immunization Laboratory the serum samples for the suspected measles cases are tested for measles IgM and rubella IgM in parallel [15] & [16]. We analyzed data from the EPI program case based measles suspected cases data from January 2007 through December 2016 (10 years) to describe the epidemiology and associated factors and to provide a baseline for the monitoring of the impact of rubella vaccine which is to be subsequently introduced into the routine immunization in 2019.

## Methods

We did a retrospective analysis of data collected during routine case based measles surveillance which is part of the routine integrated disease surveillance and Response (IDSR) in Uganda. This was also used to test rubella IgM among all measles suspected case samples.

### Study setting

The study was conducted in Uganda an East African country with a population of about 34.7 million people and consisted of 112 districts for our study. The data was for suspected measles cases from all the districts in Uganda that were detected and reported from January 2007 to December 2016.

The case definition of a suspected case of measles was any person with fever and maculopapular generalized rash and cough or coryza or conjunctivitis or any person in whom a clinician suspects measles. The community case definition was any person with fever and a rash. A confirmed case was a suspected case that tested positive for the rubella Immunoglobulin M [17].

### Data collection and serological testing

A case investigation form was filled by health worker in triplicate the third copy was submitted to the laboratory at Uganda Virus Research Institute (UVRI) with the blood specimen (serum). All collected specimen were tested for measles IgM and for rubella IgM in parallel using a standard Enzyme Immno-Assay (EIA), Enzygnost Anti-Rubella virus IgM test kit (Siemens, Marburg, Germany) [15]. The variables were those that are updated in the UNEPI case based database and included the following: Age (age group), sex (M/F), settings (rural/urban), region of residence, year of reporting, outcome of illness and whether hospitalized or not for illness.

### Data analysis

The analysis was done using STATA version 13 software. Two-sided chi-square tests for association were computed to detect differences between categorical variables such as sex, age group, setting, region, admitted or not and outcome of illness. The means of continuous variables were computed. In order to investigate the association between the outcome variable of rubella IgM serology and other variables, bivariate logistic regression was first done then multivariate logistic regression models were run for all significant factors at 95% confidence interval in the bivariate analysis. The model building strategy was not only limited to significant variables from the bivariate analysis, but also included independent variables that were considered to have a clinical and social significance for the outcome of rubella IgM positivity. The evaluation of the associations of rubella IgM sero-prevalance was based on all 15,296 cases reported during the ten year study period.

### Results: demographic characteristics of tested cases

The suspected cases were 16,551 and those that were tested for rubella were 15,296. There were 50.9% male, the largest age group was < 5 yrs. 58.3% (*n* = 8928). Most cases 10.9% were in the year 2011 and the largest number, 23.4% (*n* = 3985) from north central region of Uganda. Most 97.5% (*n* = 14,921) were from rural areas, 90.1% of the cases were not hospitalized and only ten of the cases died of the illness (cases fatality of 0.9% over ten years). Mean a**ge was** = 5.3 sd = 5 range 0 – 80 years. See further details in the Table 1.

**Table 1:** Demographic characteristics of the tested cases

Variables	n	%
Age groups: (*N* = 15,296)		
< 5 yrs	8928	58.3
5-15 yrs.	5901	38.6
> 15 yrs.	467	3.1
Sex: (*N* = 15,296)		
Male	7788	50.9
Female	7508	49.1
Region: (*N = 15,296)*		
Acholi	331	2.7
Ankole	1085	7.1
Bugisu	879	5.7
Bukedi	473	3.1
Bunyoro	750	4.9
Busoga	1510	9.9
Kampala	389	2.5
Karamoja	157	1
Kigezi	788	5.1
Lango	508	3.3
North central	3585	23.4
South central	2751	18
Teso	558	3.6
Tooro	1057	6.9
West Nile	475	2.8
Settings: (*N = 15,296)*		
Rural	14,921	97.5
Urban	375	2.5
Year: (*N* = 15,296)		
2007	1655	10.8
2008	1393	9.1
2009	1124	7.3
2010	1229	8
2011	1666	10.9
2012	1650	10.8
2013	1129	7.4
2014	1563	10.2
2015	2523	16.5
2016	1364	9
In/out patient: (*N = 15,295)*		
Hospitalized	1521	9.9
Not hospitalized	13,774	90.1
Outcome of illness: (*N = 15,296)*		
Didn’t die	15,286	99.1
Died	10	0.9

### Rubella IgM+ prevalence

Overall prevalence from all 15 regions among 15,296 cases was 27.8% (ci: 27.1–28.5). The age group with the highest prevalence was 5–15 years at 40.8% and females had a higher prevalence than the males at 28.6%, while 27.3% of all women of reproductive age (15 – 49 yrs) were rubella positive. Lango region had the highest prevalence of 43.7% with Busoga region the lowest of 17.2% and the rural areas were higher at 27.9%. The cases that were not hospitalized had a higher prevalence of 29.4%, further details in Table 2.

**Table 2:** Rubella IgM prevalence by case’s demographic characteristics

Variables	n	% (n)	95%CI
Age groups: (*N* = 15,296)			
< 5 yrs.	8928	19.5 (1740)	18.6–20.2
5-15 yrs.	5901	**40.8** (2407)	39.5–40.2
. > 15 yrs.	467	23.3 (108)	19.7–27.4
Sex: (*N* = 15,296)			
Male	7788	27 (2106)	26.1–28
Female	7508	**28.6** (2149)	27.6–29.7
Female 15- 49 yrs	322	**27.3** (88)	–
Settings (*N* = 15,296)			
Rural	14,921	**27.9** (4169)	27.2–28.7
Urban	375	22.9 (86)	18.9–27.4
Region: (*N = 15,296)*			
Acholi	331	30.2 (100)	25.5–35.4
Ankole	1085	31.1 (338)	28.5–34
Bugisu	879	34.4 (302)	31.3–37.7
Bukedi	473	32.4 (153)	28.3–36.6
Bunyoro	705	32.4 (243)	29.1–35.8
Busoga	1510	17.2 (260)	15.4–35.8
Kampala	389	22.1 (86)	18.3–19.2
Karamoja	157	24.2 (38)	18.1–31.5
Kigezi	788	40.9 (322)	37.5–44.3
Lango	508	**43.7** (222)	29.4–48.1
North central	3585	22.1 (791)	20.7–23.4
South central	2751	26.7 (733)	25–28.3
Teso	558	21 (117)	17.7–24.5
Tooro	1057	34.9 (369)	32–37.8
West Nile	475	38.1 (181)	33.8–42.6
In/out patient: (*N* = 15,295)			
hospitalized	1521	12.9 (196)	11.3–14.7
Not hospitalized	13,774	**29.4** (4059)	28.7–30.2
Outcome of illness: (*N* = 15,296)			
Died	10	**30** (3)	9.3–64.9
Alive	15,286	27.8 (4252)	27.1–28.5

**Table:** Table 3

Associated factor	Unadjusted OR (95%CI)	*p*-value	adjusted OR (95%CI)	*p*-value
Sex Female	1.1 (1.01–1.2)	**0.03**	1 (0.9–1.1)	0.254
Age: < 5 yrs	Ref		Ref	
5–15 years	2.9 (2.6–3.1)	**< 0.001**	2.5 (2.3–2.9)	**< 0.001**
> 15 years	1.3 (1.01–1.6	**0.04**	1.4 (1.1–1.8)	**0.003**
Rural setting	1.3 (1.02–1.7)	**0.03**	1 (0.5–2)	0.985
Measles negative	7.9 (6–10.3)	**< 0.001**	6.4 (4.9–8.5)	**< 0.001**
Outcome Alive	0.9 (0.23–3.5)	0.88		
Hospitalized	0.35 (0.3–0.41)	**< 0.001**	0.5 (0.4–0.6)	**< 0.001**
Busoga region(11%)	Ref			
Acholi	2.1 (1.6–2.7)	**< 0.001**	1.9 (1.5–2.6)	**< 0.001**
Ankole	2.2 (1.5–2.6)	**< 0.001**	2 (1.6–2.4)	**< 0.001**
Bugisu	2,5 (2.1–3.1)	**< 0.001**	2.1 (1.7–2.6)	**< 0.001**
Bukedi	2.3 (1.9–2.9)	**< 0.001**	2.1 (1.6–2.7)	**< 0.001**
Bunyoro	2.3 (1.9–2.8)	**< 0.001**	2.1 (1.7–2.6)	**< 0.001**
Kampala	1.4 (1.04–1.8)	**0.026**	1.4 (0.7–3)	0.336
Karamoja	1.5 (1.04–2.3)	**0.031**	1.6 (1.06–2.4)	**0.026**
Kigezi	3.3 (2.7–4)	**< 0.001**	2.6 (2.1–3.2)	**< 0.001**
Lango	3.7 (3–4.6)	**< 0.001**	3.5 (2.7–4.4)	**< 0.001**
North central	1.4 (1.2–1.6)	**< 0.001**	1.2 (1.02–1.4)	**0.031**
South central	1.7 (1.5–2)	**< 0.001**	1.5 (1.3–1.8)	**< 0.001**
Teso	1.3 (1–1.6)	**0.05**	1.3 (1–1.6)	0.055
Tooro	2.6 (2.1–3.1)	**< 0.001**	2.4 (1.2–2.9)	**< 0.001**
West Nile	2.9 (2.4–3.7)	**< 0.001**	3 (2.4–3.8)	**< 0.001**

### Ten year rubella IgM prevalence trend

**Fig. 1:**
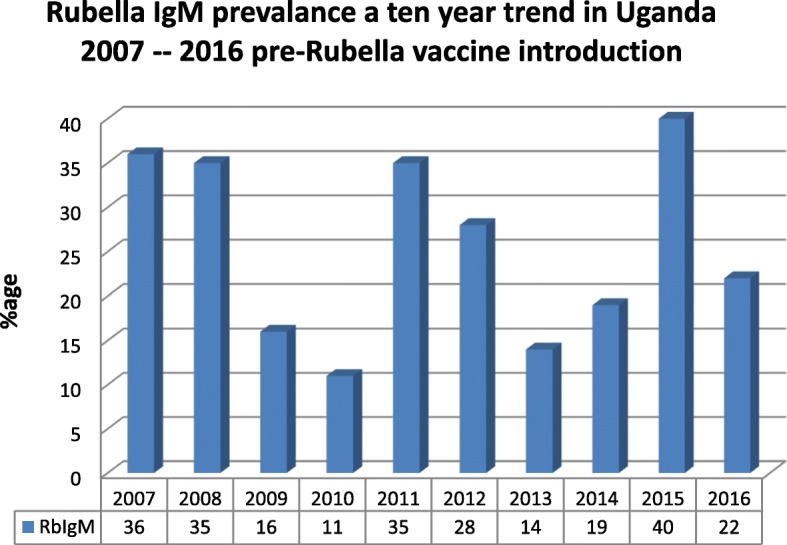
Rubella IgM prevalence a Ten year trend in Uganda 2007 to 2016 pre-rubella vaccine introduction.

Over the 10 years the prevalence was 27.8% (ci: 27.1–28.5) for all the 15,296 cases. The highest prevalence of 40% was detected in 2015 and the lowest in 2010 of 11%, The prevalence of rubella IgM peaked during the years of 2007(36%), 2008(35%), 2011(35%), 2012(28%) and 2015(40%), details are in Fig. 1.

**Fig. 2:**
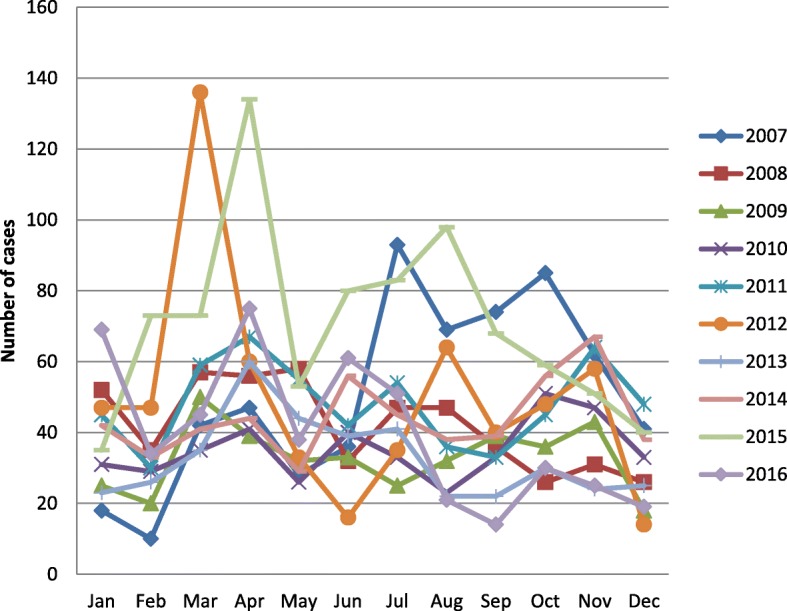
The distribution of rubella prevalence by year and month of year over 10 years.

The distribution of rubella prevalence was throughout all the month during each of the ten years with peaks in 2007, 2011, 2012 and 2015 then during the months of March, April, july and August respectively, details in Fig. 2.

### Factors associated to rubella prevalence

The factors that are significantly associated Rubella IgM prevalence are: age group 5–15 years OR 2.5 *p*-value < 0.001 and age group > 15 years OR 1.2 p-value 0.002 compared to age < 5 years. Measles negative OR 6.3 p-value < 0.001 and admitted cases OR 0.5 p-value < 0.001 were also significantly associated with rubella IgM prevalance. All the regions in Uganda except Kampala and Teso regions were associated with higher odds (1.4–3.7) of rubella prevalence greater than 11% compared to Busoga region which had the lowest prevalence. Table 3 details these results:

## Discussion

In the period January 2007 to December 2016, 16,551 suspected cases of measles were reported of which 15,296 were tested for rubella IgM [15] & [16]. Of those tested 27.8% (*n* = 4255) were rubella IgM positive from all the 15 regions in Uganda. Ninety seven point 5 % (*n* = 4147) were aged 15 years or below this is consistent with Mirambo et al. 2015 in an Africa literature review, Njeru et al. 2015 in Kenya, Mitiku, K et al. 2011 in Ethiopia and Kombich et al. 2009 in Kenya [6, 7, 18, 19] In Mozambique Dimech et al. 2016 found a prevalence of 53% which is about twice that we found in Uganda but this is still an indicator of the high transmission of rubella in the Africa region. The results are also consistent with the 3 findings cited in Kenya and Ethiopia that rubella is a disease common among under-five’s and adolescents [18] & [20]. The findings also indicate that rubella IgM prevalence is associated with increase in age compared to the children less than five years OR 2.5 p = < 0.001 and 1.4 *p* = 0.003 respectively for the age groups 5–15 and those > 15 years. These findings are consistent with Junaid et al. 2011 in Nigeria, however this was not demonstrated by Mirambo et al. 2015 in a review for Africa [6] & [20]. For the cases that tested negative for the rubella IgM depending on the effectiveness of our surveillance system the samples collected within 3 days or after 28 days of onset of the measles rash may not have sufficient detectable antigen. The WHO recommendation is that samples are taken within 28 days of the onset of the rash and this is what is done in Uganda to increase the possibility of not missing a positive test [15] & [16].

Among the measles suspected cases that were in the reproductive age group for Uganda of 15–49 years, 88 of the 322 (27%) were positive for rubella IgM this is a large percentage and an indicator that the female reproductive age group is also at risk. Although Njeru et al. 2015 had a lower prevalence in Kenya of 6% both findings are of public health importance for the two neighboring countries as this is a high risk group. Although Kenya has now introduced the rubella containing vaccine [18], Uganda has not yet hence the continuing possibility for rubella virus transmission in the East African region.

Among the cases tested for rubella IgM prevalence although there was a slight difference between the cases in the rural and urban with the rural about 2% higher this difference was not significant among the Uganda Measles suspected cases. This finding is consistent with Mirambo et al. 2015 who found a slight difference between the rural and urban areas in Nigeria [6], Mozambique and Bukina Faso but these were not significant [8].

Countries that were not or are not providing rubella containing vaccine like Uganda have reported outbreaks of rubella throughout the year which are seen mainly among under five year olds and adolescents as was reported in Uganda by, Namuwulya et al. 2015 in Uganda and Goodison et al. 2011 in Africa for the period of 2002 to 2009 [9] & [13]. These out breaks are attributed to failure to provide the rubella vaccine containing vaccine. In the WHO America region we have seen that indigenous cases of rubella were eliminated. In this region rubella transmission was interrupted and CRS eliminated by 2009 and 2015 respectively it was declared to have eliminated rubella and CRS by the World Health Organization, this is consistent with [3, 11, 21] this is further evidence that introduction of the rubella vaccine containing vaccine is crucial for the elimination of rubella infection and Congenital Rubella Syndrome.

### Limitations

Since we used the standard case definition for measles and yet we know that rubella is usually more mild in presentation than measles there is a possibility that the number of actual rubella cases may have been underestimated. About 50% of rubella cases may not present with a fever so this estimate may be lower than the actual rubella cases. Since the tested cases were suspected measles cases this data may not be inferred to the general population of Uganda. There is also a possibility that due to the varying surveillance performance over the 10 years some cases could have been missed. And also there are some socio and demographic characteristics of the cases that are not collected by the measles case form eg socio-economic status and level of education this could have left out some important associated factors.

## Conclusions

The findings demonstrate a high level of IgM prevalence and endemic distribution of rubella in all the regions of Uganda throughout the year and over all the ten years that were analyzed. These findings provide further evidence of the high prevalence of rubella among the women of reproductive age, the infants, children and adolescents. This therefore gives us the evidence that the introduction of the rubella containing vaccine is long overdue given the success it has made in the America’s and Europe.

In the context of the ongoing routine immunization performance of the past ten years and the success so far with measles, introduction of the rubella containing vaccine will enhance the control and elimination of rubella infection and Congenital Rubella Syndrome. The Uganda programme for Immunization is planning to introduce the rubella vaccine among and into the infants routine programme as a combination with the measles antigen as the MR vaccine formulation. This will immediately be followed with a catch up immunization to target adolescent females aged 9 to 15 years.

As a recommendation there is a need to start testing for IgG routinely in the future to help in the better classification of cases.

## Data Availability

All data supporting our findings are contained in the paper. There are no restrictions to data sources as it is a public national database of Uganda, however, details of the full data may be accessed through Fred Bagenda (corresponding author), Department of Community Health, Mbarara University of Science and Technology, PO Box 1410, Mbarara, Uganda, email: bagendaf@gmail.com Tel: + 256772452506.

## Abbreviations

ci: Confidence interval
CRS: Congenital Rubella Syndrome
EIA: Enzyme Immuno Assay
IDSR: Integrated Disease Surveillance and Response
IgM: Immunoglobulin M
OR: Odds Ratio
*p*-value: Probability value
UNEPI: Uganda National Expanded Programme of Immunization
UVRI: Uganda Virus Research Institute
WHO: World Health Organization
